# Supplementary material for: Spontaneous recanalization of extracranial internal carotid occlusion: A systematic scoping review
Source: PLoS One. 2025 Jul 11;20(7):e0326261. doi: 10.1371/journal.pone.0326261 (PMC12250523; doi:10.1371/journal.pone.0326261)
Supplement: S2 Appendix — (DOCX) [file pone.0326261.s002.docx]

Embase Classic+Embase <1947 to 2024 February 29>

Ovid MEDLINE(R) ALL <1946 to February 29, 2024>

EBM Reviews - Cochrane Central Register of Controlled Trials <January 2024>

1 Carotid Artery, Internal/ 46676

2 (internal adj2 carotid adj2 arter*).tw,kf. 65430

3 ica.tw,kf. 50789

4 1 or 2 or 3 113944

5 (transient adj5 (occlud* or occlusion*)).tw,kf. 11295

6 recanali*.tw,kf. 49194

7 5 or 6 60271

8 4 and 7 4504

9 exp animals/ not humans/ 17743860

10 8 not 9 3432

11 10 use medall 1368

12 limit 11 to dt=20220613-20240301 159

13 internal carotid artery occlusion/ 7338

14 (internal adj2 carotid adj2 arter* adj5 (occlusion* or occlud*)).tw. 10319

15 13 or 14 14886

16 recanalization/ 23475

17 coronary artery recanalization/ 8063

18 recanali*.tw. 48773

19 16 or 17 or 18 60656

20 15 and 19 2157

21 (transient adj5 (occlud* or occlusion*) adj6 (internal carotid arter* or ica)).tw. 103

22 20 or 21 2248

23 (exp animal/ or nonhuman/) not exp human/ 13263154

24 22 not 23 2225

25 24 use emczd 1570

26 limit 25 to dc=20220613-20240301 235

27 Carotid Artery, Internal/ 46676

28 (internal adj2 carotid adj2 arter*).tw,kw. 64470

29 ica.tw,kf. 50789

30 27 or 28 or 29 113571

31 (transient adj5 (occlud* or occlusion*)).tw,kw. 11244

32 recanali*.tw,kw. 49326

33 31 or 32 60354

34 30 and 33 4495

35 34 use cctr 173

36 limit 35 to yr="2022 -Current" 39

37 12 or 26 or 36 433

38 remove duplicates from 37 350

**Web of Science – 2024-03-01**

# Database: Web of Science Core Collection

# Entitlements:

- WOS.SCI: 1900 to 2024

- WOS.AHCI: 1975 to 2024

- WOS.ESCI: 2005 to 2024

- WOS.ISTP: 1990 to 2024

- WOS.SSCI: 1900 to 2024

- WOS.ISSHP: 1990 to 2024

# Searches:

1: TS=(internal NEAR/2 carotid NEAR/2 arter*) Results: 24788

2: TS=(ica) Results: 29525

3: #2 OR #1 Results: 48141

4: TS=(recanali*) Results: 19517

5: TS=( (transient NEAR/5 (occlud* or occlusion*))) Results: 4894

6: #4 OR #5 Results: 24324

7: #3 AND #6 Results: 1521

8: #7 Timespan: 2022-06-14 to 2024-03-01 Results: 161
