# Supplementary material for: Spontaneous recanalization of extracranial internal carotid occlusion: A systematic scoping review
Source: PLoS One. 2025 Jul 11;20(7):e0326261. doi: 10.1371/journal.pone.0326261 (PMC12250523; doi:10.1371/journal.pone.0326261)
Supplement: S1 Table — (DOCX) [file pone.0326261.s003.docx]

| Study Lead Author and Year of Publication | Lead Author | Journal Title | Year of publication | Study Design | Sample size | Recanalization sample size | Proportion of Recanalization  (if reported in study) |
| --- | --- | --- | --- | --- | --- | --- | --- |
| Positive Scans in Angiographically Proven Cases of Recanalized Cerebral Infarction | T. Irino | Stroke | 1975 | Case series | 8 | 8 | 50 |
| Angiographic Manifestations in Postrecanalized Cerebral Infarction | T. Irino | Neurology | 1977 | Retrospective cohort | 16 | 6 | 37.5 |
| Relationship Between Ophthalmic Artery Blood Flow and Recanalization of Occluded Carotid Artery. Ultrasonic Doppler Study | H. Kaneda | Stroke | 1978 | Case series | 5 | 5 | 40 |
| Spontaneous Bilateral Recanalization in Bilateral Internal Carotid Artery Occlusion | T. M. Markwalder | Stroke | 1980 | Case report | 1 | 1 | N/A |
| Bilateral Occlusion of the Extracranial Internal Carotid Artery Secondary to Closed Neck Injury | H. Muller | Neurochirurgia | 1984 | Case report | 1 | 1 | N/A |
| Transient Neurological Deficits due to Embolic Occlusion and Immediate Reopening of the Cerebral Arteries | M. Taneda | Stroke | 1985 | Case series | 1 | 1 | N/A |
| Spontaneous Recanalization of Carotid Artery Occlusion Following Non-Traumatic Dissection | G. Parenti | Italian Journal of Neurological Sciences | 1989 | Case series | 7 | 6 | N/A |
| Intracranial Haemodynamics in Patients with Spontaneous Carotid Dissection | M. Kaps | European Archives of Psychiatry and Neurological Sciences | 1990 | Case series | 6 | 6 | N/A |
| Spontaneous Dissection of the Internal Carotid Artery in 68 Patients | G. Ast | The European Journal of Medicine | 1993 | Retrospective cohort | 28 | 12 | 43 |
| Stroke in a Young Woman due to Spontaneous Bilateral Internal Carotid Dissection. A Case Report. | C. Sconocchini | Italian Journal of Neurological Science | 1994 | Case report | 1 | 1 | N/A |
| Noninvasive Monitoring of Internal Carotid Artery Dissection | W. Steinke | Stroke | 1994 | Prospective cohort study | 4 | 2 | 50 |
| Endothelial Functions in Pathophysiology of Thrombosis and Fibrinolysis: Late Spontaneous Recanalization of an Occluded Internal Carotid Artery: A Case Report | A. Manganaro | Angiology | 2002 | Case report | 1 | 1 | N/A |
| Recanalization of Acute Symptomatic Occlusions of the Internal Carotid Artery | S. H. Meves | Journal of Neurology | 2002 | Prospective cohort study | 30 | 6 | 26.7 |
| Spontaneous Recanalization of Internal Carotid Artery Occlusion Evaluated with Color Flow Imaging and Contrast Arteriography | G. Camporese | International Angiology | 2003 | Case series | 8 | 8 | 5 |
| Spontaneous Recanalization of Internal Carotid Artery Occlusion | M. N. Nguyen-Huynh | Stroke | 2003 | Case series | 2 | 2 | N/A |
| Extracranial Internal Carotid and Vertebral Artery Dissections: Angiographic Spectrum, Course and Prognosis | O. Pelkonen | Neuroradiology | 2003 | Retrospective cohort | 16 | 5 | 31.3 |
| Spontaneous Recanalization of Acute Internal Carotid Artery Occlusion | S. Calleja | Annals of Vascular Surgery | 2004 | Case report | 1 | 1 | N/A |
| Outcome in Patients with Stroke Associated with Internal Carotid Artery Occlusion | M. Paciaroni | Cerebrovascular Diseases | 2005 | Prospective cohort study | 105 | 10 | 9.5 |
| Carotid Artery Stenting in a Patient with Spontaneous Recanalization of a Proximal Internal Carotid Artery Occlusion: a Case Report | E. J. Kim | Korean Journal of Radiology | 2006 | Case report | 1 | 1 | N/A |
| Late Spontaneous Recanalization of Acute Internal Carotid Artery Occlusion | C. Klonaris | Journal of Vascular Surgery | 2006 | Case report | 1 | 1 | N/A |
| Natural history and clinical outcome of patients with documented carotid artery occlusion | S. Cheema | Irish Journal of Medical Science | 2007 | Prospective data capture, retrospective data analysis cohort | 19 | 2 | 10.5 |
| Late Spontaneous Recanalization of Internal Carotid Artery: Case Report | G. F. Saes | Jornal Vascular Brasileiro | 2007 | Case report | 1 | 1 | N/A |
| Spontaneous Recanalization of Occluded Internal Artery After Minor Stroke. The Role of Surgical Treatment | R. Adovasio | Minerva Cardioangiologica | 2008 | Case series | 3 | 3 | N/A |
| Symptomatic Late Recanalization of an Occluded Internal Carotid Artery: A Case Report and Review of the Literature | M. S. Gohel | Vascular and Endovascular Surgery | 2008 | Case report | 1 | 1 | N/A |
| Early Spontaneous Recanalization Following Acute Carotid Occlusion | K. Szabo | Journal of Neuroimaging | 2008 | Retrospective cohort | 76 | 12 | 9.2 |
| Spontaneous Recanalization of the Internal Carotid Artery Resulting in Thromboembolic Occlusion of the Ipsilateral Ophthalmic Artery and Visual Loss | M. J. Binning | Journal of Clinical Neuroscience | 2009 | Case report | 1 | 1 | N/A |
| Recanalization of Chronic Carotid Occlusion: Case Report and Review of the Literature | P. Matic | Vascular | 2009 | Case report | 1 | 1 | N/A |
| Neurosonographic Monitoring of 105 Spontaneous Cervical Artery Dissections | C. Baracchini | Neurology | 2010 | Prospective cohort study | 15 | 10 | 67 |
| Spontaneous Recanalization of an Occluded Internal Carotid Artery | P. S. Shah | Annals of Vascular Surgery | 2010 | Case report | 1 | 1 | N/A |
| Spontaneous Recanalization of Complete Internal Carotid Artery: A Clinical Reminder | S. Som | Journal of Surgical Technique and Case Report | 2010 | Case report | 1 | 1 | N/A |
| Spontaneous Recanalization of Internal Carotid Artery Occlusion | L. Tuskan-Mohar | The Middle European Journal of Medicine | 2010 | Case report | 1 | 1 | N/A |
| Spontaneous Recanalization of Chronic Internal Carotid Artery Occlusions: Report of 3 Cases | S. Buslovich | Vascular and Endovascular Surgery | 2011 | Case series | 3 | 3 | N/A |
| Benign Outcome of Objectively Proven Spontaneous Recanalization of Internal Carotid Artery Occlusion | G. Camporese | Journal of Vascular Surgery | 2011 | Prospective cohort study | 16 | 16 | 2.3 |
| Early Activation of Intracranial Collateral Vessels Influences the Outcome of Spontaneous Internal Carotid Artery Dissection | M. Silvestrini | Stroke | 2011 | Prospective cohort study | 66 | 24 | 36.4 |
| Bilateral Spontaneous Internal Carotid Artery Dissection with Both Early and Very Late Recanalization: A Case Report | E. Vicenzini | Journal of Clinical Ultrasound | 2010 | Case report | 1 | 1 | N/A |
| Late Spontaneous Recanalization of a Symptomatically Occluded Internal Carotid Artery Two Years after Extra-Intracranial Bypass | H. W. Kniemeyer | Journal of Cardiovascular Surgery | 2012 | Case report | 1 | 1 | N/A |
| Spontaneous Recanalization of the Occluded Internal Carotid Artery: a Report of Two Cases | R. Mohammadian | The Neuroradiology Journal | 2012 | Prospective cohort study | 65 | 2 | 3.1 |
| Internal Carotid Artery Occlusion: Its Natural History Including Recanalization and Subsequent Neurological Events | G. Morris-Stiff | Vascular and Endovascular Surgery | 2013 | Prospective data capture, retrospective data analysis cohort | 77 | 8 | 10.4 |
| Recurrent Strokes due to Transient Vasospasms of the Extracranial Internal Carotid Artery | S. Wopking | Case Reports in Neurology | 2013 | Case report | 1 | 1 | N/A |
| The Location of Origin of Spontaneous Extracranial Internal Carotid Artery Dissection is Adjacent to the Skull Base | J. Downer | Journal of Medical Imaging and Radiation Oncology | 2014 | Retrospective cohort | 9 | 8 | 88.9 |
| Spontaneous Multiple-Channel Recanalization of Internal Carotid Artery Occlusion with Unusual Radiological Features | T. Nozaki | Clinical Neuroradiology | 2014 | Case report | 1 | 1 | N/A |
| Late Spontaneous Recanalization of Symptomatic Atheromatous Internal Carotid Artery Occlusion | M.G. Delgado | Vascular | 2015 | Prospective cohort study | 136 | 7 | 5 |
| Spontaneous Recanalization of Chronic Occlusion of the Internal Carotid Artery | I. N. Shchanitsyn | Angiologiia i Sosudistaia Khirurgiia | 2015 | Case report | 1 | 1 | N/A |
| Factors associated with recurrent stroke and recanalization in patients presenting with isolated symptomatic carotid occlusion | D. Damania | European Journal of Neurology | 2015 | Retrospective cohort | 33 | 7 | 21.2 |
| Morphological Change of Early Spontaneous Recanalization Following Internal Carotid Artery Occlusion due to Possible Dissection | Z. Zhou | Chinese Medical Journal | 2016 | Case report | 1 | 1 | N/A |
| Spontaneous Recanalization After Carotid Artery Dissection: The Case for an Ultrasound-Only Monitoring Strategy | S. Lumsden | Methodist Debakey Cardiovascular Journal | 2017 | Case report | 1 | 1 | N/A |
| Acute Cerebral Infarction After Spontaneous Recanalization of  Extracranial Internal Carotid Artery Atherosclerotic  Occlusion: Two Case Reports and Literature Review | K. Jin | International Journal of Clinical and Experimental Medicine | 2018 | Case series | 2 | 2 | N/A |
| Spontaneous Recanalization of a Chronic Internal Carotid Artery Occlusion | H. Tejada Meza | Neurologia | 2018 | Case report | 1 | 1 | N/A |
| Prognosis of Patients with Late Spontaneous Recanalization of the Atherosclerotic Occlusion of Internal Carotid Arteries: A Pilot Case Series | H. L. Wu | Experimental and Therapeutic Medicine | 2018 | Prospective cohort study | 36 | 3 | 8.3 |
| Functional Outcome after Symptomatic Internal Carotid Artery Occlusion | I. Stanescu | Balneo Research Journal | 2019 | Case report | 1 | 1 | N/A |
| New Cortical Spot Cerebral Infarction Out of Border Zone in ICA Occlusion Suggests Recanalization: A Case Report | H. Inoue | NMC Case Report Journal | 2022 | Case report | 1 | 1 | N/A |
| Spontaneous Recanalization of Internal Carotid Artery Occlusion: A Case Report | S. Y Zhang | The Neurologist | 2023 | Case report | 1 | 1 | N/A |
| Internal Carotid Artery Dissection with Different Interventions and Outcomes: Two Case Reports | C. Zheng | Journal of International Medical Research | 2023 | Case report | 1 | 1 | N/A |
