# Supplementary material for: Spontaneous recanalization of extracranial internal carotid occlusion: A systematic scoping review
Source: PLoS One. 2025 Jul 11;20(7):e0326261. doi: 10.1371/journal.pone.0326261 (PMC12250523; doi:10.1371/journal.pone.0326261)
Supplement: S2 Table — (DOCX) [file pone.0326261.s004.docx]

| Study category | Age (years, median ± standard deviation) | % males (median) |
| --- | --- | --- |
| Cohort studies | 66.2 ± 10.7  (n = 5 studies) | 65  (n = 6 studies) |
| Case reports/series | 54 ± 13.7  (n = 29 studies) | 100  (n = 35 studies) |
