# Supplementary material for: Spontaneous recanalization of extracranial internal carotid occlusion: A systematic scoping review
Source: PLoS One. 2025 Jul 11;20(7):e0326261. doi: 10.1371/journal.pone.0326261 (PMC12250523; doi:10.1371/journal.pone.0326261)
Supplement: Attachment — Submitted filename: PLOSOne Reviewer Comments_final.docx [file pone.0326261.s006.docx]

**Comments to the Author**

**Reviewer #1**

Comment #1: “The authors conducted a scoping review of studies reporting spontaneous recanalisation after ICA occlusion. This subject is interesting and refers to an entity that is otherwise underestimated in the literature. Though interesting the study presents some major limitations. The main limitation is the heterogeneity regarding the included studies. Case series most often report spontaneous recanalisation in individuals who come with symptom recurrence or new events. On the other hand, prospective studies or RCTs have a more "strict" protocol of follow up imaging. Combining these disparate approaches to estimate prevalence might be confusing.”

Response: We agree with the reviewer that case series may be biased towards patients with symptomatic presentations. Indeed, our study reveals that median prevalence in case series alone was 40.0%, whereas in cohort studies alone only 21.2%. For this reason, we had separated the outcomes by type of study. As discussed in our limitations, there was significant heterogeneity in reporting recanalization events, particularly amongst case series where cases were identified individually without an overarching protocol for identifying recanalizations. We have included this is in the limitations (Pg. 9-10, Lines 214-217). We have also added a sentence in our limitations section acknowledging that a prospective cohort study with prespecified imaging protocols is ultimately needed to confirm these findings and have discussed this limitation in our discussion (Pg. 10, Lines 223-226).

Comment #2: “Another drawback is that it refers to ICA occlusion in general. This combines both occlusion caused by a dissection and occlusion due to atherothrombosis. These are entirely distinct entities with well documented natural history. They will, in my opinion, be handled and reported separately. More specifically, the occurrence of spontaneous recanalization of an occlusion due to an underlying atherosclerotic plaque is a rare event for which there is a dearth of information and recommendations.

Response: The reviewer raises a valid point. We have added quantitative information about the number of dissection studies in our results section (Pg. 4, Lines 105-108). We have included the prevalence findings quantitatively in the results section (Pg. 4, Lines 114-116). Timeline results have been added qualitatively to Fig 2, and the corresponding legend has been updated (Pg. 6, Line 132).

**Reviewer #2**

Comment #1: **“**The possibility of spontaneous recanalization of an occluded internal carotid artery (ICA) has been known for years. The challenge lies not in the fact of recanalization itself but in determining the management strategy after its detection and its impact on the patient's neurological condition.”

Response: We appreciate and agree with this comment. However, we do not agree that this is necessarily a commonly held opinion amongst stroke specialists. Moreover, there is a lack of understanding within the stroke community as to the prevalence of spontaneous carotid recanalization; we wish to highlight the comment from reviewer #1 from above: “This subject is interesting and refers to an entity that is otherwise underestimated in the literature”. We believe despite the methodological limitations of a systematic scoping review, our study is a necessary first step to advance our understanding of this concept. Once we have a better understanding of the prevalence of spontaneous recanalization, we can estimate the sample sizes and inform study designs to investigate management strategies. As mentioned in our limitation section, a prospective cohort study will be the next step in confirming or refuting the findings in this scoping review (Pg. 10, Lines 223-226).

Comment #2: “The article lacks basic data on the neurological status of patients at the time of occlusion diagnosis, the onset of neurological symptoms during the occlusion, and after the confirmation of recanalization. These are key pieces of information when discussing this topic. Please establish the neurological history of the patients analyzed.”

Response: We agree with the reviewer regarding the importance of clinical information during the acute event, as it speaks to the potential for bias in patients that are symptomatic at presentation (see Reviewer #1 Comment #1). Unfortunately, a scoping review approach does not allow for a granular assessment of clinical presentation and neurological history; we were limited to the information published in the source literature. However, our intention is to use the information within this systematic review to design a prospective cohort study that will capture neurological status at initial diagnosis and at present imaging timelines.

Comment #3: “Page 11; Line 117. The material includes acute, subacute, and chronic ICA occlusions. The mechanism of spontaneous recanalization differs across these subgroups, as do the prognosis and treatment approaches. Do the authors have data that would allow for a separate analysis of these subgroups?”

Response: Unfortunately, the source data does not specify the exact mechanism of spontaneous recanalization. There is data however about the mechanism of initial occlusion, as we have outlined above in our response to Reviewer 1 (Comment #2). Etiology of occlusion will be an important piece of information that we will build into our prospective cohort study, as we have outlined in our discussion (Pg. 10, Lines 223-226).

Comment #4: “Page 12; Line 134 Imaging of extracranial ICA occlusion in the context of spontaneous recanalization requires information on the patency of this artery in the intracranial segments as well. DUS examination is performed to assess the C1 segment. In CTA, the site of restored circulation in the ICA is often not visible. The reference examination for assessing the length of ICA occlusion is selective angiography from the contralateral artery. Do the authors have data regarding the length of ICA occlusion? Do the authors have data on whether the spontaneous recanalization manifested as, for example, a string sign, complete recanalization, or significant stenosis in the C1 segment?”

Response: Unfortunately, due to the study design, we are limited to the data provided in our source material. Very few studies have information on the morphology of the recanalization, and those that report it are exclusively case series/reports. However, we have added this point to our limitation section (Pg. 10, Lines 218-221).

Comment #5” “Page 13; Line 154 The surgical management of patients with spontaneous recanalization requires information on the nature of the stenosis. Did the intervention methods, such as CEA, CAS, or lack thereof, pertain to patients after acute or chronic occlusions? What types of stenoses were they? What were the outcomes of the surgical treatments?”

Response: The timing of intervention (CEA and CAS) varied significantly amongst studies and were implemented for both acute occlusions (as soon as 3 days post-occlusion) and chronic occlusions (up to 10 yrs post-occlusion). Unfortunately, there is very limited information regarding the degree of stenosis. The majority of studies only stated “stenosis”, with occasional qualitative description (ex. “critically stenosed”) as clarification.

Not every study reported outcomes: out of 18 studies which involved an intervention, only 11 studies reported neurological outcomes. Out of these 11 studies, 9 reported a “favourable” outcome in the way that there was no acute complication from surgery. Additionally, they report that there was a lack of neurological symptoms following a specific time period after intervention (ex. ranges from 1 mo – 1 yr). Two studies described poor outcomes: one case report mentioned rapid deterioration of neurologic condition following stent, but no further details (Inoue 2022), another reported hyperperfusion syndrome following CEA (Shah 2010). We have added this data to our results section (Pg. 7, Lines 161-167). Unfortunately, due to the inherent heterogeneity of our source data set, there is no pre-established interval of follow-up. As mentioned in our revised limitations, a prospective cohort study would better address this.

Comment #6: “Page 15; Line 207 Conclusions On what basis did the authors conclude that patients with spontaneous recanalization who underwent surgery benefited from this approach?”

Response: We believe the reviewer is referring to the statement in the conclusion, “some of which may benefit from follow-up imaging and/or revascularization”. We agree this statement needs to be tempered given the limitations discussed above. We acknowledge that it is difficult to say whether revascularization is indicated and will lead to better outcomes. Our intention was to highlight the current guidelines for revascularization of significantly stenosed carotids and suggested that recanalized carotids may require a similar approach. But we take the point that one cannot necessarily extrapolate data from acute symptomatic carotids to a spontaneously recanalized carotid. We have therefore changed the conclusion to say, “The clinical significance of spontaneous recanalization is uncertain, requiring further investigation into potential benefits from follow-up imaging and/or revascularization.” (Pg. 10, Lines 230-234).
