# Supplementary material for: Spontaneous recanalization of extracranial internal carotid occlusion: A systematic scoping review
Source: PLoS One. 2025 Jul 11;20(7):e0326261. doi: 10.1371/journal.pone.0326261 (PMC12250523; doi:10.1371/journal.pone.0326261)
Supplement: Attachment — Submitted filename: PLOSOne Reviewer Comments_Round 2_final.docx [file pone.0326261.s007.docx]

**PLOSOne Reviewer Comments Round #2**

**Reviewer #1:**

All previous comments were addressed by the authors in the revised manuscript.
The manuscript has been significantly enhanced.

Response: We thank the reviewer for their time and feedback.

**Reviewer #3:**

The authors have responded to the multiple reviewer questions and concerns, particularly regarding to the limitations of the source data.

Response: We thank the reviewer for their time and helpful comments.

**Reviewer #4:**

This systematic scoping review of spontaneous recanalization following extracranial ICA occlusion has important limitations. The significant heterogeneity in reporting and identifying ICA recanalization events, due to the lack of a standard timeline for surveillance imaging after carotid occlusion, limits the strength of the conclusions. The majority of recanalizations were identified secondary to symptomatic recurrent clinical events, potentially biasing the results towards symptomatic cases, particularly in case reports. There is also a possibility that some cases reported as occlusion on non-invasive imaging may have been near occlusions with trickle flow. Additionally, the scoping review design provides limited data on the exact morphology of spontaneous recanalization, such as thrombus length and radiological markers. The study combines occlusions caused by dissection and atherothrombosis, which are distinct entities with different natural histories that should ideally be analyzed separately. A prospective cohort study with prespecified imaging protocols is ultimately needed to reduce heterogeneity and confirm these findings.

Response: We agree fully with the reviewer. It is indeed our plan to conduct a methodologically rigorous prospective cohort study to address exactly these limitations. Our scoping review provides the justification in doing so, and we see it as our first step.

**Reviewer #5**

**Major points**

Comment #1: Zhang and colleagues present a literature review to inform an important clinical question: is occluded internal carotid artery really a benign finding i.e. safe to not offer intervention as one would for a high grade stenosis? This is an interesting and valuable attempt at quantifying the prevalence of spontaneous extracranial ICA recanalization, but it has some important weaknesses that are largely addressed by the original reviewers. I note that I am not one of the original reviewers but am considering the comments of and responses to the original reviewers in my assessment. The concerns I have are really the same. These are addressed, I think, with modest effort given the apparent limitations of the underlying data, which is largely to say that the authors could do very little to address these concerns. Thus, my foremost recommendation would be to further temper the discussion of the manuscript to a level appropriate for data that is, at best, suggestive of the conclusions drawn. I acknowledge that it is not the fault of the authors that the underlying data may be of poor quality or not particularly informative for clinical use, but it is the responsibility of the authors to find diamonds in the rough that will justify their proposed prospective cohort study. There is a substantial amount of work to be done for this manuscript to be interpretable.

Response: We completely appreciate the reviewers’ assessment of our project. We also recognize the reviewer was not one of the original reviewers so we appreciate the fresh insight and value the time taken. We hope that this scoping review will provide the foundation from which to launch a methodologically rigorous cohort study to address the limitations of this dataset. In this context, we have done our best to address the points below (within the limitations of the design) and have tempered the manuscript accordingly.

Comment #2: I have a hard time believing that there is no data on neurological status available in any of the manuscripts reviewed. Any information would be helpful here and would be of value to answer the fundamental question: does it matter whether an artery spontaneously recanalizes? Yes, such a patient would then meet criteria for intervention if symptomatic in a technical sense but a chronic occlusion from atherosclerotic disease versus a dynamic dissection flap is an extremely different pathology with different risk of presenting future neurologic complications. The authors at least once say most presentations for recanalization were at symptom onset with no further information provided. This is contradictory and confusing and undermines my confidence in the manuscript.

Response: We apologize for being unclear in our first round of responses. When we said we could not provide neurological status, we were referring to the specific presenting details such as clinical syndrome (aphasia, side of hemiplegia, NIHSS, etc.) or other physical examination findings, the details of which varied significantly from article to article. But we did not mean to imply we could not determine “symptomatic” vs “asymptomatic”; the reviewer is correct that this information was provided in some articles, and is indeed highly relevant. We have now added to the Results (under the section “Timeline of Recanalization”), *“Forty-nine studies (14 cohort studies, 35 case studies) reported the timing of imaging demonstrating recanalization after initial occlusion, either after symptomatic presentation (6 studies reported stroke, 3 reported TIA, 2 reported both TIA & stroke) or through routine check-up (38 studies, asymptomatic).”* Note that the previous full-text specified 42 studies which reported the timing of imaging, rather than 49. We would like to be transparent and admit this was an error in the text that was found while addressing this comment. This has no bearing on subsequent analysis as all analysis completed was on these 49 studies. However, we have now corrected the number in the text.

Comment #3: As a consequence of point 1), this study lacks the granularity I would expect of a scoping review that included 53 studies, even if the majority of them were case studies.

Response: We appreciate the reviewer’s comment. We have included more granularity in our data, including reported the number of symptomatic (TIA vs stroke) vs asymptomatic status at time of recanalization, as well as clinical status following recanalization in both revascularized and non-revascularized cases. Additional specific changes can be found in the responses to the comments below.

Comment #4: I do think this review article can be salvaged but it requires the addition of a section in the results section discussing the above-mentioned points, and major rewriting of discussion and restructuring of figures. Consolidate figures 2 and 3 into one figure, use the additional figure space to illustrate some of the more specific data reviewers 1 and 2 had asked about.

Response: We thank the reviewer for this suggestion. We have consolidated Figures 2 and 3 into one figure, and the following caption was added to the Results section (Pg. 20): “Fig 2. Histogram of timing of imaging between occlusion and recanalization identification. Red circles = studies including dissection. A) Distribution amongst cohort studies. B) Distribution of individual participants in case reports/series.”

Reviewer #5 comment regarding our response to Reviewer #1’s original comment: How can you estimate prevalence from a case series if cases are selected based on the occurrence of spontaneous recanalization? The denominator of this calculation is nebulous at best.

Response: Thank you for this important comment. Our calculations of prevalence were based only on studies which themselves reported prevalence of spontaneous recanalization over a larger sampled population of non-recanalized occlusions. This is reflected in the Method section (under Data collection and synthesis of results) which states, *“Prevalence of recanalization was calculated if the paper reported on a population of both non-recanalized and recanalized cases.”* Overall, there were only 3 case series which reported a prevalence. In the first case series (Irino 1975), the initial ICA occlusion population was 8 patients, 4 of which recanalized, for an overall prevalence of 50%. In Kaneda 1978, there were 5 occlusion patients, 2 of which recanalized (prevalence of 40%). Finally, in Camporese 2003, 8 of the initial 160 ICA occlusion patients recanalized, for a prevalence of 5%. The remainder of the case series/reports did not specify a larger occlusion population; therefore, they were not included in the prevalence calculation, as prevalence could not be reported.

Reviewer #5 comment regarding Reviewer #1’s comment #2: The difference between recanalization and after occlusion due to dissection vs atherosclerotic plaque was not sufficiently discussed.

Response: Thank you for bringing this to our attention. We had attempted to address this issue in our response to Reviewer #1 through the following additions: *“We have added quantitative information about the number of dissection studies in our results section (Pg. 4, Lines 105-108). We have included the prevalence findings quantitatively in the results section (Pg. 4, Lines 114-116). Timeline results have been added qualitatively to Fig 2, and the corresponding legend has been updated (Pg. 6, Line 132).”* We believe our study design cannot reliably distinguish between occlusions due to plaque vs dissection because we are dependent on the descriptions of authors from our source articles: we cannot assess the radiology ourselves, we cannot comment on the radiological expertise of the original authors, and we cannot measure the heterogeneity in radiological assessments between studies. While the paper would undoubtedly be strengthened by these additions, it would require access to individual patient-level data and would be outside the scope of a scoping review. In the future prospective cohort study we hope will be justified by this study, we will specifically address this issue.

Reviewer #5’s comment on reviewer #2’s comment #1: Question about impact on patient’s neurological condition. This is a comment that could have and should have been addressed in more detail here and is reiterated in greater detail by this reviewer in comment #2. This is the fundamental underlying question of this work and needs to be reasoned through and answered as much as is possible with the available data.

Response: Thank you for this comment. To offer more detail on patients’ neurological statuses, we have now added the numbers of symptomatic vs. asymptomatic status when recanalization was detected.

Reviewer #5’s comment on reviewer #2’s comment #3: I appreciate the researchers including this reference to planned prospective cohort study. However, simply suggesting a possible future study is insufficient. At the very least, I would expect a detailed and candid discussion of the things that cannot be addressed in this review.

Response: Thank you, we are happy to add specific elements that a cohort study can and should address, and we are open to suggestions from the reviewer to inform our future study design. In addition to whether an occlusion is acute, subacute or chronic, a prospective study can attempt to answer whether the etiology of an occlusion is thought to be atherosclerotic, embolic or dissection. Moreover, we can perform more sophisticated imaging to identify trickle flow and describe the morphology of the carotid after recanalization. A prospective study with standardized imaging protocols can also determine the best timelines for reimaging after a confirmed carotid occlusion. Finally, we can collect baseline demographics, clinical history and examination findings, and assess for clinical events during a follow-up period. While the details of our future cohort study are still in development (and will remain as such pending the final publication of this manuscript which will inform its design), we have now added to our limitation paragraph: *“Overall, while this scoping review provides an important introduction to quantifying spontaneous recanalization, a prospective cohort study with prespecified imaging protocols is ultimately needed to address the limitations of this scoping review, reduce heterogeneity and confirm these findings. Specifically, a prospective cohort study should have standardized clinical and radiological assessments with longitudinal follow-up, and utilize sophisticated imaging such as dynamic CT-angiography and MR-angiography to confirm occlusion status, describe carotid morphology following recanalization, and attempt to distinguish atheroembolic occlusion from dissection.”* Again, we are open to recommending additional components to a cohort study design at the reviewers’ suggestion. We fully intend to use this scoping review to justify the need to perform a cohort study.

Reviewer #2 comment on reviewer #2’s comment #4: Agree with report results of commenting on recanalization morphology in the results section, this would be interesting. Given alleged 40% prevalence from the case series evaluated, I would be very surprised that zero of them had e.g. angiographic descriptions.

Response: Thank you for this interesting comment and agree about the importance of including angiographic descriptions. We wish to clarify that although there are descriptions of angiographic features of the occlusions/recanalizations in the reviewed literature, we are unfortunately limited by the descriptions given in the source article. There is significant heterogeneity in level of detail of description. For example, in Irino et al.’s (1977) paper, they have multiple descriptors of the post-recanalization angiographic findings, including arterial narrowing, capillary blush, and residual stenosis. This is in contrast with Wu et al. (2018), who only described the recanalizations as a “string sign” on DSA. Finally, in studies by Kaneda et al. (1978), Adovasio et al. (2008), and Mohammadian et al. (2012), there were no descriptions of the artery besides “recanalization”. Accordingly, we have now added to the Results, *“There was significant heterogeneity with respect to angiographic descriptions of recanalization ranging from a higher level of detail (ex: arterial narrowing, capillary blush, and residual stenosis), minimal detail (ex: string sign), and no detail beyond “recanalization”.”*

Reviewer #5’s comment on reviewer #2’s comment #5: This information, regarding acuity/chronicity, is interesting and valuable and should be included in the main body of the text.

Response: Thank you, we agree and have added the additional information. This section (Treatment and Outcomes) now includes, *“These interventions were completed on spontaneous recanalizations of both acute and chronic occlusions, with a time frame ranging from 3 days to 10 years post-occlusion”* and, *“In these cases, the former intervention was completed 4 days post-occlusion, while the latter was completed at 8 months”* and, “*Where revascularization was either not reported or not done, 18 studies did not describe clinical outcomes following spontaneous recanalization, 14 reported asymptomatic outcomes, 2 reported mild neurological deficits, 2 reported moderate-severe neurological deficit, and 2 reported death.”*

Reviewer #5’s comment on reviewer #2’s comment #6: There are studies that comment on the significance of spontaneous recanalization that can and should be cited here.

Response: We thank the reviewer for their comment. We have provided further support for the significance of spontaneous recanalization and its potential indications for intervention in our discussion section: *“Moreover, there is evidence in the literature in a shift of management for these cases. In a qualitative review by Lall et al., the authors’ discussion suggest that surgical revascularization tailored to the lesion and patient would be reasonable in both asymptomatic and symptomatic recanalizations (Lall et al., 2021). Similarly, Delgado et al. recommend intervention in symptomatic recanalizations, citing that occlusions may not be as benign as previously thought (Delgado et al. 2015)”.* We are open to citing additional sources at the reviewers’ discretion.

Lines 66-67: There must have been more exclusion criteria to lose so many (96% to be exact) of your harvested studies in the search process. If additional criteria were used, please specify these explicitly in the text

Response: Thank you for this comment. We worked with a pair of health sciences librarians with a specialty in devising systematic/scoping review search strategies to create our search criteria. They advised that we use a very broad and deliberately over-inclusive search strategy so as to ensure that we were in a position to capture all available articles and not to accidentally exclude relevant articles. Consequently, we expected many exclusions. There were no additional criteria used. Most importantly, our search strategy specified any recanalization rather than specifically spontaneous recanalization in order to be as inclusive as possible. As such, the initial search yielded many manuscripts which included surgical and interventional recanalization and revascularization of intra- and extracranial vessels (i.e. endovascular thrombectomy, endarterectomy, carotid angioplasty and stenting), rather than spontaneous recanalization of ICA occlusions. We have included the list of manuscripts screened out at the abstract level in the supplemental data.

Lines 100-105: I am extremely suspicious that patient age and sex were not recorded in so many of these studies. These are very basic demographics and the lack of recording, suggest poor-quality evidence. This alone may be disqualifying for this manuscript if fundamental sociodemographics are not available from the studies.

Response: Thank you for this comment, and we hope to allay the reviewer’s suspicions with the following clarification. The reviewer is correct that sex and age were indeed recorded in studies, and we wish to clarify what we were attempting to say: the cohort studies that we described as not reporting sex or age in fact reported an overall distribution of both parameters across a population that included patients beyond those with just ICA occlusion, such as patients with vertebral artery occlusion or ICA stenosis without occlusion. As such, while these demographics were reported, we could not determine the age and sex of the specific patient sample in our study (i.e. ICA occlusion only). We have changed the wording in the methods to better explain (in Study and Patient Characteristics), *“Participant age was reported in the ICA occlusion population in 5 cohort studies and 29 case studies, with a median age of 66.2 ± 10.7 years and 54 ± 13.7, respectively (S2 Table). Thirty-five case studies reported sex in the ICA occlusion population, whereas only 6 cohort studies did so.”*

Line 106: The meaning of "specified dissection" is not clear to me? I.e., 17 studies listed cases of dissection or 17 included it as a category regardless of whether those participants were included or not.

Response: Thank you for this comment. To explain, 17/34 studies reported dissection as an etiology. Of these 17 studies, 15 included only dissection patients, whereas 2 studies (Meves 2002, Paciaroni 2005) included both dissection and atheroembolic cases. The remaining 17 studies either did not specify an etiology, or only described atheroembolic cases. We have clarified this in the main text, *“A total of 34 studies reported the etiology of occlusion, 17 of which specified dissection as a possible etiology (9 case series, 8 cohort studies). Of these 17 dissection studies, 15 included only patients with dissection, whereas 2 studies included both patients with dissection and atheroembolic occlusions. The remaining 17 studies either did not specify an etiology, or only described atherosclerotic or cardioembolic etiologies.”*

Lines 119-120: Please comment on the relative frequencies of symptomatic presentation vs routine check-up diagnoses.

Response: Thank you for this comment. We have now added data about the frequency of symptomatic presentation vs. routine diagnosis. The text now reads: *“Forty-nine studies (14 cohort studies, 35 case studies) reported the timing of imaging demonstrating recanalization after initial occlusion, either after symptomatic presentation (6 studies reported stroke, 3 reported TIA, 2 reported both TIA & stroke) or through routine check-up (38 studies, asymptomatic).”*

Line 122-3: Please re-word. “Time frame in cohort studies” is total time of follow up or is time to recanalization?

Response: Thank you for the suggestion. We have changed the wording to “timing of imaging demonstrating recanalization” to help clarify.

Line 142: Without additional imaging modalities or could one patient have more than one imaging modality? If yes, were both counted in this review or how was decided which one was counted?

Response: Occlusion and recanalization were often confirmed using more than one modality in the same patient or cohort (ex. occlusion initially identified with ultrasound, then confirmed with angiography or CTA/MRA). If more than one modality was used, all were quantified rather than just one; therefore, it is not a 1 modality to 1 study ratio (ie modalities are not mutually exclusive). As a result, the sum of the different modalities does not equal to 100%, as shown in the Imaging results section and Table 1.

Line 158: Are these participants? Of how many total?

Response: Thank you for your question. Yes, these are individual participants, as mentioned in the “Treatment and Outcomes” section: *“At the individual level, the majority of the participants’ medical treatment was undescribed. However, in both pre- and post-recanalization, antiplatelet-only treatment was most common (n = 191, n = 37, respectively).”* This was also clarified in the caption of Table 2B.

Lines 161-162: Lack of neurological data is again concerning re: underlying data. I think for a neurovascular pathology with intervention criteria defined by symptomatology, this again may be a disqualification of the manuscript.

Response: Thank you for this comment. We have included neurological data about participants. Symptomatic status has now been included: *“Forty-nine studies (14 cohort studies, 35 case studies) reported the timing of imaging demonstrating recanalization after initial occlusion, either after symptomatic presentation (6 studies reported stroke, 3 reported TIA, 2 reported both TIA & stroke) or through routine check-up (38 studies, asymptomatic).”*

Line 179: I do not think the authors can say this likely occurs in more than 20% of cases. I think this is statement that must be tempered substantially if not entirely removed. The number of excluded studies and the opacity of the denominator in these data do not allow the authors to say this. The authors could rephrase to a “We calculate an estimated XX% incidence of recanalization based on our extremely low quality data”.

Response: Thank you for this comment. We appreciate the need to further temper our conclusions, and admit that the data in this space is limited. But we wish to gently express that we take issue with the reviewer’s words, “extremely low quality data.”

As mentioned in our response above, the reason for the high number of excluded studies is related to the deliberately highly inclusive search strategy, as advised by our two expert librarians who are both highly experienced with systematic reviews (Dewar and Shorr). Moreover, our calculation of prevalence was done in consultation with expert methodologists and statisticians at the Ottawa Method Centre. Our methodology is sound, and the protocol with the search strategy was published a priori in accordance to open science principles. While we agree it is appropriate to say scoping reviews are a relatively low level of evidence and hypothesis generating at best, we also believe it is inappropriate to say “extremely low quality data”. Accordingly, we have changed the wording in the discussion to be significantly more tempered: *“recanalization likely may occur in approximately 20% of cases and may be identified within 6 months after occlusion”* and, *“However, the scoping review design has important methodological limitations and these findings should be considered hypothesis generating pending validation in a prospective fashion.”*

Lines 180-182: The authors must temper this statement. They are suggesting a high cost and potential intervention (which is a non-zero risk of major procedural complications up to and including stroke, MI, or death) based on low quality data.

Response: Thank you for this comment. We have endeavoured to moderate our conclusions.

Line 184: The majority of studies in this review were also case studies. The authors should make it clear that their estimate of prevalence is also mostly derived from case studies.

Response: Thank you for the opportunity to clarify. While the reviewer correctly points out case series likely overestimate prevalence, it is not the case that our prevalence was calculated mostly from case series. In fact, the majority of the studies used to calculate prevalence were cohort studies (17 cohort studies and 3 case series). While the overall prevalence of 24.0% listed in the results was calculated as a median of all reported studies (20 in total), the specific line in the discussion the reviewer has highlighted, “recanalization may occur in approximately 20% of cases” refers to the cohort studies only. We provide these details in the Results, including the prevalence of 21.2% from 17 cohort studies alone, the 40.0% prevalence from the case series alone, as well as the combined prevalence of 24.0% in the cohort + case series. For the Discussion, we opted to highlight the more conservative prevalence derived from only cohort studies, and further tempered the point by stating it as, “approximately 20%”.

Lines 189-191: You should cite directly from the trial not just from a secondary review.

Response: Thank you for this comment. Given the secondary review described the initial findings to “carotid territory” strokes, whereas the initial PROACT studies concerned MCA strokes specifically, we opted to remove this section of the discussion altogether for greater clarity and accuracy.

Line 199: “Given that most of the cases included in this study presented symptomatically either at occlusion or recanalization…” Suggests strongly that the authors do have this data. What were the symptoms? If only from case series, that is fine but is critical and would be informative.

Response: Thank you for this comment, we have added the following text to try to clarify this issue: *“Forty-nine studies (14 cohort studies, 35 case studies) reported the timing of imaging demonstrating recanalization after initial occlusion, either after symptomatic presentation (6 studies reported stroke, 3 reported TIA, 2 reported both TIA & stroke) or through routine check-up (38 studies, asymptomatic).”*

Line 203: “there is case report-level evidence” change to "there are case reports of successful revascularization..."

Response: Thank you for the suggestion. We have made the change.

Line 220: “the majority of cases used the diagnostic gold standard of angiography“ As per Tables, the majority of patients were diagnosed via ultrasound unless multiple modalities were counted more than once.

Response: Thank you for this comment. As detailed in our response to Line 142 above, all diagnostic modalities were counted, even when multiple modalities were used for a single patient or cohort. As such, when identifying occlusion, both ultrasound and angiography were used in over 50% of cases and were not mutually exclusive. The majority of occlusions were indeed confirmed with conventional angiography.

Line 229: 21.2% - What is this number derived from?

Response: Thank you for this question. As discussed in more depth to the comment on Line 184, this number is the prevalence in the cohort studies only. A median was calculated from a sample of 17 cohort studies, as outlined in the results section. As above, the prevalences were calculated in consultation with expert methodologists at the Ottawa Methods Centre: <https://www.ohri.ca/ottawamethodscentre/about.aspx?trk=public_post-text>

Table 2: In part A, the numbers for pre- (n=54) and post-recanalization (n=53) don't match. Where does the missing study belong?

Response: Thank you for this comment. There are two discrepancies which account for the total studies not equaling 53. Firstly, one study which specified “no treatment” was not initially included out of error, and has now been added to Table 2, and the results section.

Secondly, within one study, there was sometimes multiple types of treatment reported, resulting in a ratio other than 1:1 study to treatment. For example, in Buslovich 2011, out of the 3 total patients in the case series, one received anticoagulation before recanalization, while the other two patients did not have any described treatment. In this such case, both anticoagulation and “undescribed” were counted for this one study. In the pre-recanalization group, multiple treatments were described in 2 studies (undescribed + anticoagulation), resulting in 2 extra studies being added to both the undescribed and anticoagulation categories, for a total of 55 studies. In the post-recanalization group, there was only one study that reported multiple treatments (undescribed + combo) resulting in 1 extra study being added to the combo and undescribed categories, for a total of 54 studies.

Figure 1: Records excluded are very high. Please specify why these records were excluded.

Response: As described above, this was a deliberate choice to be overinclusive in the search strategy and include all recanalization, not just spontaneous recanalization of a previously occluded carotid.

Figure 2: This is not a good graph because there is so much overlap between datapoints and is very challenging to interpret. There is also a tremendous amount of white space in the figures. Consider alternative designs such as cumulative incidence functions.

Response: We thank the reviewer for their comment. We have removed Figure 2 (dot plot) and merged Figure 2 and 3 into one figure.

Figure 3: Maybe I am misunderstanding but it seems that this is the same information that is presented in figure 2 and thus is totally unnecessary. If these are different, it needs to be made clearer how these are different data.

Response: We agree with the reviewer and have merged the figures as above.
