# Supplementary material for: Spontaneous recanalization of extracranial internal carotid occlusion: A systematic scoping review
Source: PLoS One. 2025 Jul 11;20(7):e0326261. doi: 10.1371/journal.pone.0326261 (PMC12250523; doi:10.1371/journal.pone.0326261)
Supplement: Attachment — Submitted filename: Reviewer comments_final.docx [file pone.0326261.s008.docx]

**PLOSOne Reviewer Comments Round #3**

**Reviewer #4:**

This scoping review addresses an important clinical question; however, several specific limitations require revision.

Comment #1: First, the heterogeneity in imaging modalities and timing of recanalization assessment significantly weakens conclusions. Clarify how multiple imaging modalities per patient were handled statistically to avoid double-counting.

Response: Thank you for this comment. We agree that the heterogeneity of the data included in this scoping review remains high, and such emphasize that this scoping review acts as a hypothesis-generating review, with the intention of guiding a future prospective cohort study. With respect to the multiple imaging modalities. Occlusion and recanalization were often confirmed using more than one modality in the same patient or cohort (ex. occlusion initially identified with ultrasound, then confirmed with angiography or CTA/MRA). If more than one modality was used, all were quantified rather than just one; therefore, it is not a 1 modality to 1 study ratio (i.e. modalities are not mutually exclusive). As a result, the sum of the different modalities does not equal to 100%, as shown in the Imaging results section and Table 1. For example, in the paper by Kaps et al. (1990), occlusion was identified with both angiography and ultrasound doppler, therefore one study was counted towards both angiography and doppler. We opted to quantify the imaging modalities this way in order to most accurately capture the full breadth of data provided in the sample and avoid bias by choosing one modality over the other.

Comment #2: Second, combining dissections with atherothrombotic occlusions is problematic given distinct natural histories; separate analyses or subgroup sensitivity analyses are recommended.

Response: We agree with the reviewer, and have clarified and presented prevalence data separated by etiology in our results section: “When broken down by etiology, median prevalence was 39.7% (IQR 32.8 – 54.3%) in dissection studies vs. 8.75% (IQR 5.70 – 10.5%) in non-dissection studies.” (Pg. 5, lines 116-118). Additionally, we show the data of timing of imaging of recanalization in dissection vs. non-dissection studies in our updated Figure 2, where dissection data is now highlighted with red circles.

Comment #3: Third, the prevalence calculation from case series is questionable due to unclear denominators; restrict prevalence estimates to cohort studies only.

Response: We thank the reviewer for raising this point, and we have accordingly removed the prevalence data from the case series in our results section: “An overall proportion of recanalization events following carotid occlusion was calculated for 17 cohort studies, for a median prevalence of 21.2% (IQR 9.2 – 37.5%)” (Pg. 5, lines 115-116).

Comment #4: Fourth, neurological outcomes and symptomatology at recanalization are inadequately detailed; explicitly report symptomatic versus asymptomatic presentations.

Response: Thank you for this comment. We had previously attempted to address this issue in our response to Reviewer #5 in the previous round of revisions by adding the number of symptomatic vs. asymptomatic status when recanalization was detected: *“Forty-nine studies reported the timing of imaging demonstrating recanalization after initial occlusion, either after symptomatic presentation (6 studies reported stroke, 3 reported TIA, 2 reported both) or through routine check-up (38 studies, asymptomatic).”* Pg. 5, lines 121-123.

Comment #5: Finally, the discussion overstates findings; temper conclusions to reflect the hypothesis-generating nature of data.

Response: We have further tempered our conclusion, which now reads: “In this study, we demonstrate that spontaneous recanalization following occlusion of extracranial ICA is possible in up to 21.2% of patients. Our findings warrant caution in the context of acute ICA occlusion and require prospective validation in a large sample using predetermined repeat imaging timepoints.” We have replaced the stronger term “occurs” with “is possible” and have removed the comment about imaging and revascularization. Moreover, the abstract and discussion were previously similarly tempered in a revision.
