# Supplementary material for: Spontaneous recanalization of extracranial internal carotid occlusion: A systematic scoping review
Source: PLoS One. 2025 Jul 11;20(7):e0326261. doi: 10.1371/journal.pone.0326261 (PMC12250523; doi:10.1371/journal.pone.0326261)
Supplement: Attachment — Submitted filename: Reviewer_comments_final_auresp_4.docx [file pone.0326261.s009.docx]

**PLOS One Reviewer Comments Round #4**

**Reviewers #1 & 3**: responses were accepted and no further questions as of January 20^th^, 2025.

**Reviewer #5**: no further questions as of March 24th, 2025.

**Reviewer #4 Dr. Robert J. Chen (March 24^th^, 2025):**

The authors have made commendable revisions in response to prior concerns; however, some issues persist.

Comment #1: While imaging heterogeneity is acknowledged, the handling of multiple modalities per patient remains insufficiently clarified—quantifying per study rather than per patient risks duplication bias.

Response: We have added information in the results section clarifying how the various modalities were quantified at the study level: *“In some cases, multiple modalities were used to measure both occlusion and recanalization in the same study. As such, to avoid bias and most accurately capture the full range of modalities used, we report the number of studies that used each modality. Insufficient detail was provided to report the number of patients in which each modality was used.”* (Pg. 7, lines 140-143).

Unfortunately, not all source studies provided sufficient detail on which modalities were used in which individuals, specifically in cohort studies. Four out of 17 cohort studies did not specify the imaging modality at the individual patient level (Damania 2016, Delgado 2015, Morris-Stiff 2015, and Paciaroni 2005); therefore, we are limited to quantifying at the study level. While we completely appreciate why Dr. Chen is asking for this information, these data require patient-level meta-analysis that is beyond the scope of this study.

Comment #2: Although dissection and non-dissection etiologies are now separated, the categorization could benefit from consistent application and clearer definitions across analyses.

Response: First and foremost, we are extremely appreciative of the time and effort reviewer Dr. Chen has invested in improving our manuscript. We also understand that Dr. Chen has identified an important point about dissections that we have incorporated into the design of a prospective study that is informed by this systematic review: namely, that the definition of dissection needs to be standardized a priori. But for this study, after careful consideration and consultation with our Ottawa Method Centre colleagues, we feel we are now at risk of straying too far from our original published protocol to attempt to impose a standardized definition of dissection on already published work. Moreover, while the intention is to provide clarity on distinct etiological causes of occlusion, we do not believe that further clarification would be beneficial to the average reader.

Comment #3: Prevalence estimates have appropriately excluded case series; this correction improves methodological rigor.

Response: We thank the reviewer for their comment.

Comment #4: Neurological outcomes and symptom status at recanalization are partially addressed but remain underreported—explicit patient-level counts and clinical context are needed.

Response: Thank you for this comment. Unfortunately, a patient-level pooled analysis is beyond the scope of this paper. As a result, we do not have patient level data about symptoms and outcomes. Nevertheless, we have added more detail to the instances where neurological deficits were described after spontaneous recanalization occurred in a new table (see below) and added this table to our results section: *“Forty-nine studies (14 cohort studies, 35 case studies) reported the timing of imaging demonstrating recanalization after initial occlusion, either after symptomatic presentation (6 studies reported stroke, 3 reported TIA, 2 reported both) or through routine follow up (38 studies, asymptomatic) (Table 1).”* (Pg. 6). Because this is not a patient-level meta-analysis, we are limited in our descriptions to what the source material provided.

| **Lead author & year of publication** | **Symptoms at recanalization** | **Stroke vs. TIA** |
| --- | --- | --- |
| Saes 2007 | Amaurosis fugax to the left, aphasia, buccal angle deviation to the left and temporary worsening of motor deficit | TIA |
| Gohel 2008 | Intermittent L eye monocular blindness | TIA |
| Camporese 2011 | Two patients with non-focal symptoms, one with dysarthria, one with “transient ischemic attack” (no further details) | TIA |
| Mohammadian 2012 | One patient with an episode of  right side hemiparesis and dysarthria lasting  a few hours eight months later (TIA), one with right side hemiplegia and  global aphasia (stroke) | TIA, stroke |
| Wopking 2013 | One patient with a short episode with paresis of the right  hand and aphasia (stroke), one with (recurrent episodes with transient right-sided hemiparesis and aphasia (TIA) | TIA, stroke |
| Jin 2018 | Two patients had sudden onset of right limb weakness | Stroke |
| Wu 2018 | Two patients with recurrent stroke (no further details) | Stroke |
| Inoue 2022 | Worsened right paralysis | Stroke |
| Zhang 2023 | Transient right upper extremity weakness and worsening aphasia. | Stroke |
| Binning 2009 | Sudden loss of vision in the right eye | Stroke |
| Buslovich 2011 | Transient episodes of right-hand weakness and right amaurosis fugax | Stroke |

Comment #5: Finally, despite some moderation, elements of the discussion continue to imply clinical applicability beyond the scope of this review; further tempering is warranted.

Response: Thank you for your comment. This review is only intended to inform a prospective study, and we did not intend to imply that imply its results should dictate clinical management. We have searched carefully through the manuscript and removed an additional statement from the abstract, “whether intervention is needed, and when any intervention should occur”.

We do not believe the introduction makes management claims. Neither do the methods and results.

In the discussion, we have reworded the opening paragraph to the following: “These results suggest that recanalization does occur with sufficient frequency to be clinically relevant. However, this scoping review cannot address key questions such as whether routine surveillance is warranted, how often it should be performed, or whether revascularization should be pursued when recanalization is detected; these issues merit investigation in future prospective studies.”

The third paragraph summarizes the matter of revascularization as published in the literature – this is important because it contextualizes our study. We feel that omitting this section would be doing a disservice to our readers. Nowhere in this paragraph do we suggest a management change. Rather, we again highlight the timeline of possible recanalization (see Results section: Timeline of recanalization, Pg. 5).

We then conclude with a neutral data statement and an appeal for validation.

We wish to emphasize that we agree with Dr. Chen: we are not trying to imply clinical applicability, and if there are any parts of our manuscript that say otherwise, we are open to revising them accordingly. For this reason, we are open to Dr. Chen suggesting specific textual edits to further temper the text if required; in this scenario, we will formally acknowledge Dr. Chen’s contributions in a revision.

Comment #6: Overall, the manuscript addresses an important gap, but further revision is necessary to ensure analytical precision and appropriate interpretation of results.

Response: Thank you for this comment and we have endeavoured to address the comments raised above.
