# Supplementary material for: Spontaneous recanalization of extracranial internal carotid occlusion: A systematic scoping review
Source: PLoS One. 2025 Jul 11;20(7):e0326261. doi: 10.1371/journal.pone.0326261 (PMC12250523; doi:10.1371/journal.pone.0326261)
Supplement: Attachment — Submitted filename: Reviewer_comments_final_auresp_5.docx [file pone.0326261.s010.docx]

**PLOS One Reviewer Comments Round #5**

**Reviewers #1 & 3**: responses were accepted and no further questions as of January 20^th^, 2025.

**Reviewer #5**: no further questions as of March 24th, 2025.

**Journal Requirements:** Please review your reference list to ensure that it is complete and correct. If you have cited papers that have been retracted, please include the rationale for doing so in the manuscript text, or remove these references and replace them with relevant current references. Any changes to the reference list should be mentioned in the rebuttal letter that accompanies your revised manuscript. If you need to cite a retracted article, indicate the article’s retracted status in the References list and also include a citation and full reference for the retraction notice.

Response: We have gone through our bibliography and all included references and have not found evidence of article retraction.

**Reviewer #4 Dr. Robert J. Chen (March 24^th^, 2025):**

This scoping review addresses an important topic but has several methodological and interpretive limitations.

Comment #1: The authors pool heterogeneous studies (cohorts and case reports) without adequate weighting or bias assessment, making the reported “prevalence” (median ~21%) potentially misleading.

Response: We have removed the word prevalence and replaced it with “proportion”. We have also removed it from the title.

Comment #2: Inclusion criteria and definitions (true occlusion vs high-grade stenosis, and recanalization threshold) should be clarified.

Response: We have included these clarifications in our Methods section: *“Stenosis and recanalization were defined as any degree of blockage ≤99%, as defined in the source studies. Additionally, all etiologies of occlusion were included, including dissection.”* (Pg. 3-4, lines 72-74).

Comment #3: Different imaging modalities (Doppler US, CTA, angiography) and variable follow-up intervals are not accounted for, raising detection bias. Statistical reporting relies on unweighted descriptive metrics without confidence intervals or heterogeneity analysis. There is no appraisal of study quality or publication bias. Case reports inherently bias timing and frequency; the claim that recanalization is more common than thought seems overstated. Study-level data (sample sizes, recanalization counts) should be reported, and a meta-analytic or weighted approach considered to provide confidence intervals. Overall conclusions must be tempered given these limitations.

Response: We thank the reviewer for raising these limitations. Our Ottawa Methods Centre (internationally recognized in their work with systematic reviews) have advised that heterogeneity analysis or appraisal of study quality are not required for scoping reviews and were therefore not included in the published protocol. In response to the suggestions, we have provided a supplementary table with the additional information requested above. Moreover, we further tempered our abstract and discussion:

Abstract: *“Spontaneous recanalization of an occluded extracranial carotid artery may occur, and possibly within 6 months after documented occlusion. However, clear data are lacking regarding a standard approach to imaging or treatment of patients with occluded carotid arteries.” (Pg. 2, Lines 34-36).*

Discussion/limitations:

*“These results suggest that recanalization does occur with sufficient frequency to be clinically relevant. However, this scoping review cannot address key questions such as whether routine surveillance is warranted, how often it should be performed, or whether revascularization should be pursued when recanalization is detected; these issues merit investigation in future prospective studies.”* (Pg. 10, lines 217-221)

*“Second, the majority of recanalizations were not identified secondary to a symptomatic recurrent clinical event. For this reason, proportion and timeline findings were reported separately between case series and cohort studies.”* (Pg. 11, lines 259-260).

*“Finally, due to the nature of the scoping review, more rigorous statistical reporting was not implemented which limit the conclusions of this study.”* (Pg. 11, lines 267-269).

**S1 Table. Reference and study-level recanalization counts of 53 included studies**

| Study Lead Author and Year of Publication | Lead Author | Journal Title | Year of publication | Study Design | Sample size | Recanalization sample size | Proportion of Recanalization  (if reported in study) |
| --- | --- | --- | --- | --- | --- | --- | --- |
| Positive Scans in Angiographically Proven Cases of Recanalized Cerebral Infarction | T. Irino | Stroke | 1975 | Case series | 8 | 8 | 50 |
| Angiographic Manifestations in Postrecanalized Cerebral Infarction | T. Irino | Neurology | 1977 | Retrospective cohort | 16 | 6 | 37.5 |
| Relationship Between Ophthalmic Artery Blood Flow and Recanalization of Occluded Carotid Artery. Ultrasonic Doppler Study | H. Kaneda | Stroke | 1978 | Case series | 5 | 5 | 40 |
| Spontaneous Bilateral Recanalization in Bilateral Internal Carotid Artery Occlusion | T. M. Markwalder | Stroke | 1980 | Case report | 1 | 1 | N/A |
| Bilateral Occlusion of the Extracranial Internal Carotid Artery Secondary to Closed Neck Injury | H. Muller | Neurochirurgia | 1984 | Case report | 1 | 1 | N/A |
| Transient Neurological Deficits due to Embolic Occlusion and Immediate Reopening of the Cerebral Arteries | M. Taneda | Stroke | 1985 | Case series | 1 | 1 | N/A |
| Spontaneous Recanalization of Carotid Artery Occlusion Following Non-Traumatic Dissection | G. Parenti | Italian Journal of Neurological Sciences | 1989 | Case series | 7 | 6 | N/A |
| Intracranial Haemodynamics in Patients with Spontaneous Carotid Dissection | M. Kaps | European Archives of Psychiatry and Neurological Sciences | 1990 | Case series | 6 | 6 | N/A |
| Spontaneous Dissection of the Internal Carotid Artery in 68 Patients | G. Ast | The European Journal of Medicine | 1993 | Retrospective cohort | 28 | 12 | 43 |
| Stroke in a Young Woman due to Spontaneous Bilateral Internal Carotid Dissection. A Case Report. | C. Sconocchini | Italian Journal of Neurological Science | 1994 | Case report | 1 | 1 | N/A |
| Noninvasive Monitoring of Internal Carotid Artery Dissection | W. Steinke | Stroke | 1994 | Prospective cohort study | 4 | 2 | 50 |
| Endothelial Functions in Pathophysiology of Thrombosis and Fibrinolysis: Late Spontaneous Recanalization of an Occluded Internal Carotid Artery: A Case Report | A. Manganaro | Angiology | 2002 | Case report | 1 | 1 | N/A |
| Recanalization of Acute Symptomatic Occlusions of the Internal Carotid Artery | S. H. Meves | Journal of Neurology | 2002 | Prospective cohort study | 30 | 6 | 26.7 |
| Spontaneous Recanalization of Internal Carotid Artery Occlusion Evaluated with Color Flow Imaging and Contrast Arteriography | G. Camporese | International Angiology | 2003 | Case series | 8 | 8 | 5 |
| Spontaneous Recanalization of Internal Carotid Artery Occlusion | M. N. Nguyen-Huynh | Stroke | 2003 | Case series | 2 | 2 | N/A |
| Extracranial Internal Carotid and Vertebral Artery Dissections: Angiographic Spectrum, Course and Prognosis | O. Pelkonen | Neuroradiology | 2003 | Retrospective cohort | 16 | 5 | 31.3 |
| Spontaneous Recanalization of Acute Internal Carotid Artery Occlusion | S. Calleja | Annals of Vascular Surgery | 2004 | Case report | 1 | 1 | N/A |
| Outcome in Patients with Stroke Associated with Internal Carotid Artery Occlusion | M. Paciaroni | Cerebrovascular Diseases | 2005 | Prospective cohort study | 105 | 10 | 9.5 |
| Carotid Artery Stenting in a Patient with Spontaneous Recanalization of a Proximal Internal Carotid Artery Occlusion: a Case Report | E. J. Kim | Korean Journal of Radiology | 2006 | Case report | 1 | 1 | N/A |
| Late Spontaneous Recanalization of Acute Internal Carotid Artery Occlusion | C. Klonaris | Journal of Vascular Surgery | 2006 | Case report | 1 | 1 | N/A |
| Natural history and clinical outcome of patients with documented carotid artery occlusion | S. Cheema | Irish Journal of Medical Science | 2007 | Prospective data capture, retrospective data analysis cohort | 19 | 2 | 10.5 |
| Late Spontaneous Recanalization of Internal Carotid Artery: Case Report | G. F. Saes | Jornal Vascular Brasileiro | 2007 | Case report | 1 | 1 | N/A |
| Spontaneous Recanalization of Occluded Internal Artery After Minor Stroke. The Role of Surgical Treatment | R. Adovasio | Minerva Cardioangiologica | 2008 | Case series | 3 | 3 | N/A |
| Symptomatic Late Recanalization of an Occluded Internal Carotid Artery: A Case Report and Review of the Literature | M. S. Gohel | Vascular and Endovascular Surgery | 2008 | Case report | 1 | 1 | N/A |
| Early Spontaneous Recanalization Following Acute Carotid Occlusion | K. Szabo | Journal of Neuroimaging | 2008 | Retrospective cohort | 76 | 12 | 9.2 |
| Spontaneous Recanalization of the Internal Carotid Artery Resulting in Thromboembolic Occlusion of the Ipsilateral Ophthalmic Artery and Visual Loss | M. J. Binning | Journal of Clinical Neuroscience | 2009 | Case report | 1 | 1 | N/A |
| Recanalization of Chronic Carotid Occlusion: Case Report and Review of the Literature | P. Matic | Vascular | 2009 | Case report | 1 | 1 | N/A |
| Neurosonographic Monitoring of 105 Spontaneous Cervical Artery Dissections | C. Baracchini | Neurology | 2010 | Prospective cohort study | 15 | 10 | 67 |
| Spontaneous Recanalization of an Occluded Internal Carotid Artery | P. S. Shah | Annals of Vascular Surgery | 2010 | Case report | 1 | 1 | N/A |
| Spontaneous Recanalization of Complete Internal Carotid Artery: A Clinical Reminder | S. Som | Journal of Surgical Technique and Case Report | 2010 | Case report | 1 | 1 | N/A |
| Spontaneous Recanalization of Internal Carotid Artery Occlusion | L. Tuskan-Mohar | The Middle European Journal of Medicine | 2010 | Case report | 1 | 1 | N/A |
| Spontaneous Recanalization of Chronic Internal Carotid Artery Occlusions: Report of 3 Cases | S. Buslovich | Vascular and Endovascular Surgery | 2011 | Case series | 3 | 3 | N/A |
| Benign Outcome of Objectively Proven Spontaneous Recanalization of Internal Carotid Artery Occlusion | G. Camporese | Journal of Vascular Surgery | 2011 | Prospective cohort study | 16 | 16 | 2.3 |
| Early Activation of Intracranial Collateral Vessels Influences the Outcome of Spontaneous Internal Carotid Artery Dissection | M. Silvestrini | Stroke | 2011 | Prospective cohort study | 66 | 24 | 36.4 |
| Bilateral Spontaneous Internal Carotid Artery Dissection with Both Early and Very Late Recanalization: A Case Report | E. Vicenzini | Journal of Clinical Ultrasound | 2010 | Case report | 1 | 1 | N/A |
| Late Spontaneous Recanalization of a Symptomatically Occluded Internal Carotid Artery Two Years after Extra-Intracranial Bypass | H. W. Kniemeyer | Journal of Cardiovascular Surgery | 2012 | Case report | 1 | 1 | N/A |
| Spontaneous Recanalization of the Occluded Internal Carotid Artery: a Report of Two Cases | R. Mohammadian | The Neuroradiology Journal | 2012 | Prospective cohort study | 65 | 2 | 3.1 |
| Internal Carotid Artery Occlusion: Its Natural History Including Recanalization and Subsequent Neurological Events | G. Morris-Stiff | Vascular and Endovascular Surgery | 2013 | Prospective data capture, retrospective data analysis cohort | 77 | 8 | 10.4 |
| Recurrent Strokes due to Transient Vasospasms of the Extracranial Internal Carotid Artery | S. Wopking | Case Reports in Neurology | 2013 | Case report | 1 | 1 | N/A |
| The Location of Origin of Spontaneous Extracranial Internal Carotid Artery Dissection is Adjacent to the Skull Base | J. Downer | Journal of Medical Imaging and Radiation Oncology | 2014 | Retrospective cohort | 9 | 8 | 88.9 |
| Spontaneous Multiple-Channel Recanalization of Internal Carotid Artery Occlusion with Unusual Radiological Features | T. Nozaki | Clinical Neuroradiology | 2014 | Case report | 1 | 1 | N/A |
| Late Spontaneous Recanalization of Symptomatic Atheromatous Internal Carotid Artery Occlusion | M.G. Delgado | Vascular | 2015 | Prospective cohort study | 136 | 7 | 5 |
| Spontaneous Recanalization of Chronic Occlusion of the Internal Carotid Artery | I. N. Shchanitsyn | Angiologiia i Sosudistaia Khirurgiia | 2015 | Case report | 1 | 1 | N/A |
| Factors associated with recurrent stroke and recanalization in patients presenting with isolated symptomatic carotid occlusion | D. Damania | European Journal of Neurology | 2015 | Retrospective cohort | 33 | 7 | 21.2 |
| Morphological Change of Early Spontaneous Recanalization Following Internal Carotid Artery Occlusion due to Possible Dissection | Z. Zhou | Chinese Medical Journal | 2016 | Case report | 1 | 1 | N/A |
| Spontaneous Recanalization After Carotid Artery Dissection: The Case for an Ultrasound-Only Monitoring Strategy | S. Lumsden | Methodist Debakey Cardiovascular Journal | 2017 | Case report | 1 | 1 | N/A |
| Acute Cerebral Infarction After Spontaneous Recanalization of  Extracranial Internal Carotid Artery Atherosclerotic  Occlusion: Two Case Reports and Literature Review | K. Jin | International Journal of Clinical and Experimental Medicine | 2018 | Case series | 2 | 2 | N/A |
| Spontaneous Recanalization of a Chronic Internal Carotid Artery Occlusion | H. Tejada Meza | Neurologia | 2018 | Case report | 1 | 1 | N/A |
| Prognosis of Patients with Late Spontaneous Recanalization of the Atherosclerotic Occlusion of Internal Carotid Arteries: A Pilot Case Series | H. L. Wu | Experimental and Therapeutic Medicine | 2018 | Prospective cohort study | 36 | 3 | 8.3 |
| Functional Outcome after Symptomatic Internal Carotid Artery Occlusion | I. Stanescu | Balneo Research Journal | 2019 | Case report | 1 | 1 | N/A |
| New Cortical Spot Cerebral Infarction Out of Border Zone in ICA Occlusion Suggests Recanalization: A Case Report | H. Inoue | NMC Case Report Journal | 2022 | Case report | 1 | 1 | N/A |
| Spontaneous Recanalization of Internal Carotid Artery Occlusion: A Case Report | S. Y Zhang | The Neurologist | 2023 | Case report | 1 | 1 | N/A |
| Internal Carotid Artery Dissection with Different Interventions and Outcomes: Two Case Reports | C. Zheng | Journal of International Medical Research | 2023 | Case report | 1 | 1 | N/A |
